# Solitary Fibrous Tumors of the Pleura

**DOI:** 10.7759/cureus.12998

**Published:** 2021-01-30

**Authors:** Maimona Iftikhar Ali, Ghulam Aftab, Ali Akram

**Affiliations:** 1 Biochemistry, Akhtar Saeed Medical College, Lahore, PAK; 2 Pulmonary Medicine, Saint Peter's University Hospital/Rutgers Robert Wood Johnson Medical School, New Brunswick, USA; 3 Internal Medicine, Lahore Medical and Dental College, Lahore, PAK; 4 Internal Medicine, Wright Center for Graduate Medical Education, Scranton, USA

**Keywords:** solitary fibrous tumors, neoplasm, pleura, pleural mass, biopsy

## Abstract

Solitary fibrous tumors of the pleura (SFTP) are rare neoplasms. We present a case of a 53-year-old female presenting to the pulmonary clinic after an incidental finding of a right-sided chest wall tumor on a chest X-ray. A CT scan of the chest showed a pleural-based right upper lung mass; a biopsy of the mass was consistent with a solitary fibrous tumor.

## Introduction

Tumors of the pleura may present as either localized or diffuse pleural thickening. Solitary fibrous tumors, initially described in 1931, are localized [1]. These tumors can arise anywhere in the body; however, they most commonly arise in the pleura. Solitary fibrous tumors of the pleura (SFTP) are mesenchymal in origin and represent less than 5% of pleural tumors [2]. 

## Case presentation

**Figure 1:**
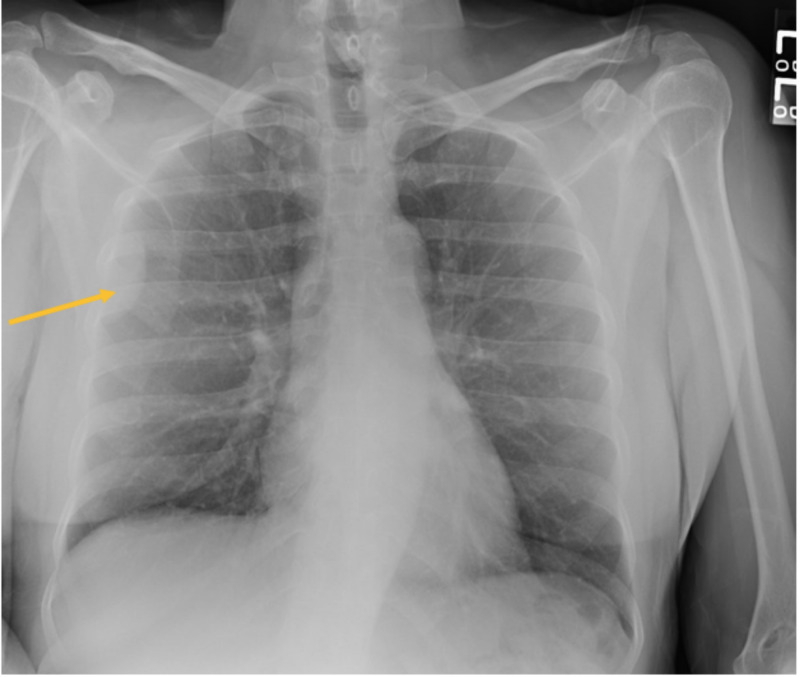
Chest X-ray showing solitary fibrous tumor.

**Figure 2:**
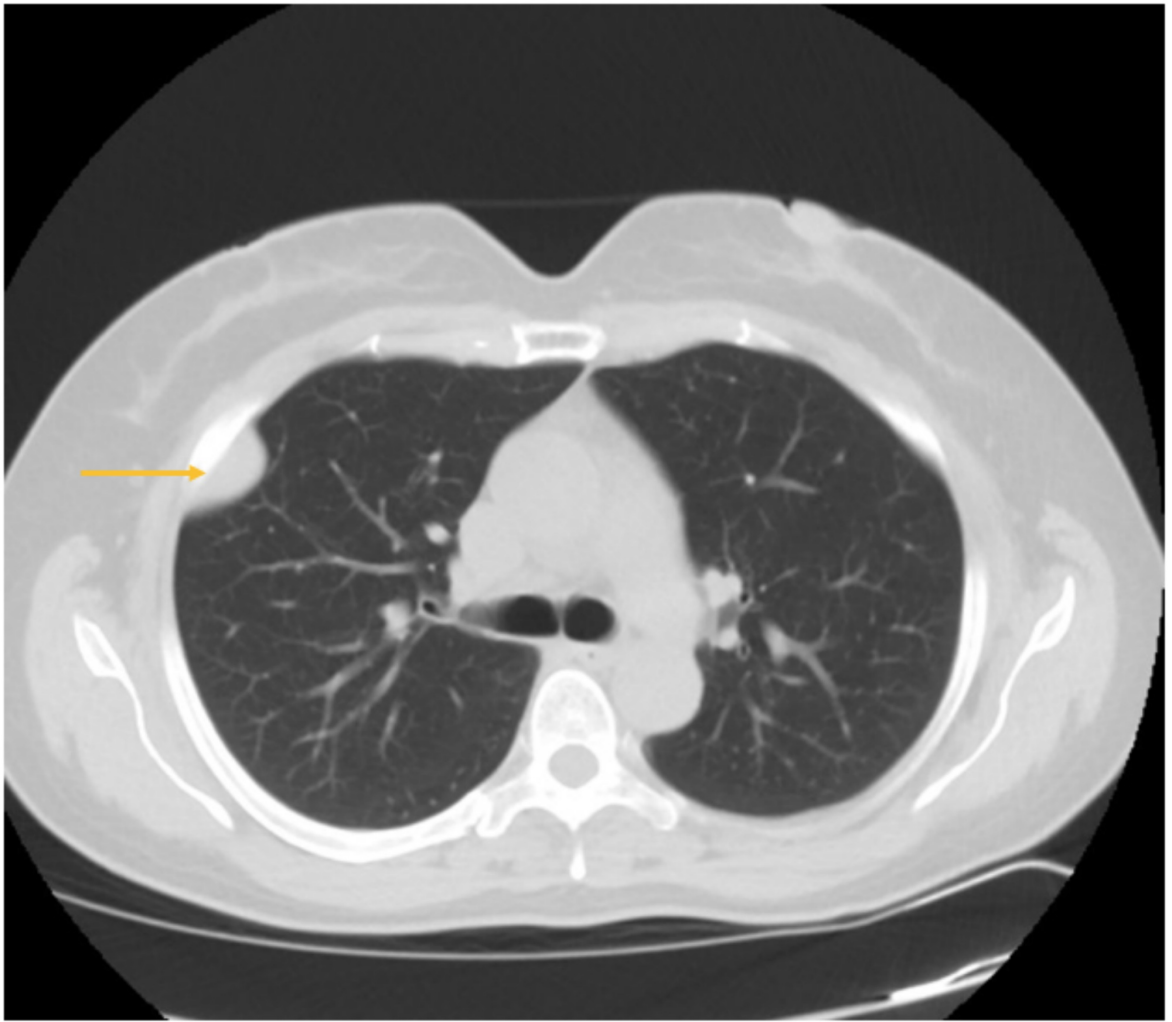
CT scan showing solitary fibrous pleural tumor.

Our patient is a 53-year-old female, who initially presented to the hospital’s ED after a motor vehicle accident. During her evaluation, she had a chest X-ray performed, which showed an incidental right-sided chest wall tumor, as shown in Figure 1. This was followed by a CT thorax, which showed a 3.3 cm x 1.5 cm pleural-based mass-like density, as shown in Figure 2. 

After this, an ultrasound of the chest was performed to evaluate for hemothorax; however, the ultrasound imaging did not demonstrate any pleural fluid collection. 

On follow-up in the clinic, the patient reported right-sided pleuritic chest pain. She did not report any cough, dyspnea, or history of previous pulmonary disease (including pneumonia or tuberculosis). She was a lifelong nonsmoker and had no asbestos exposure. 

**Figure 3:**
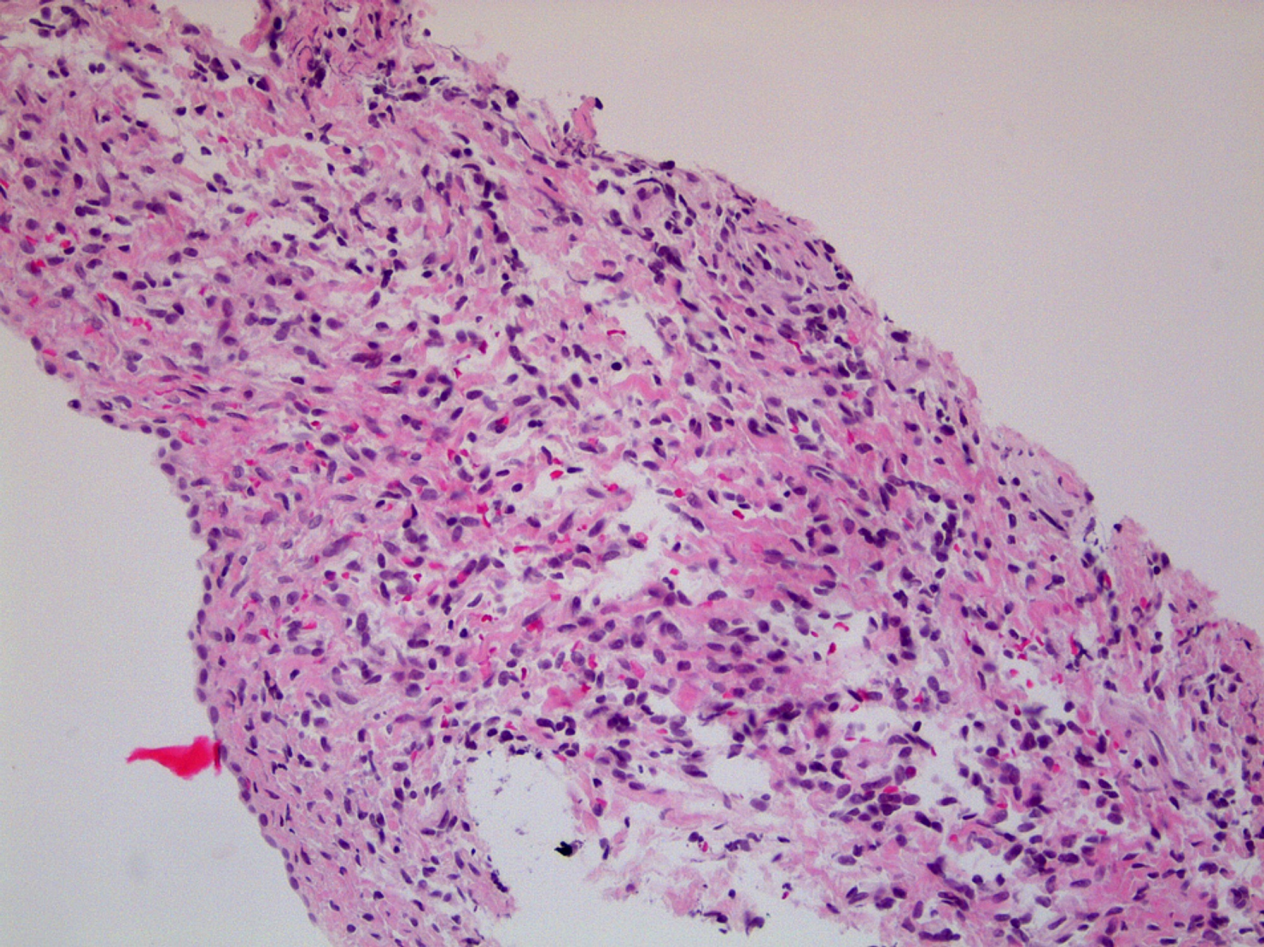
Spindles with interposed collagen.

**Figure 4:**
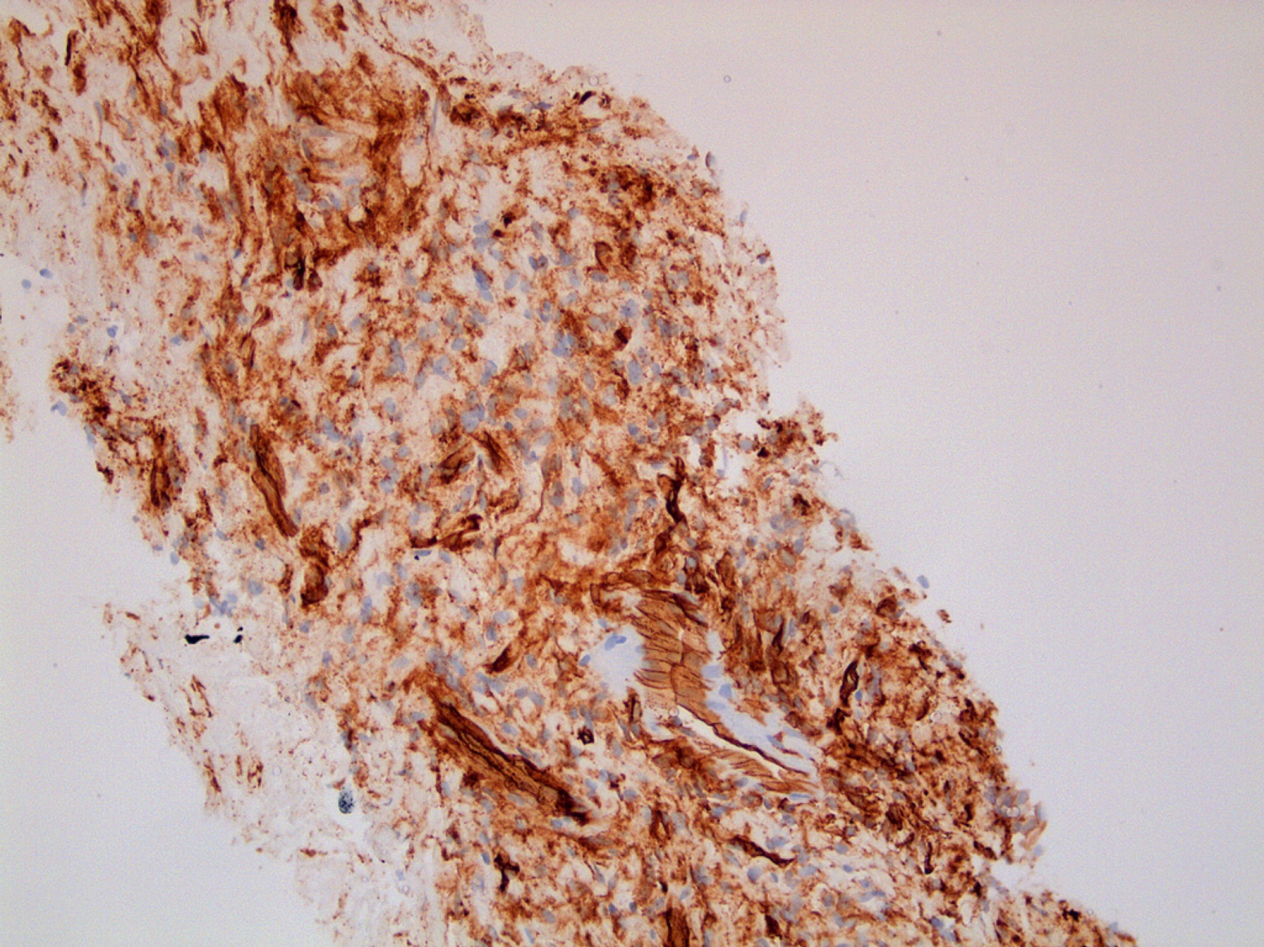
Immunohistochemical staining showing positive CD34.

The patient was referred for a CT-guided biopsy of the pleural tumor -- the biopsy showed spindle cell proliferation, with features indicating SFTP. Figure 3 shows the spindle cells with interposed collagen. Additionally, immunohistochemical stains of the biopsy sample were positive for CD34, CD99, and vimentin. The positive CD34 stain is shown in Figure 4. 

The patient was referred to cardiothoracic surgery for tumor resection. 

## Discussion

Solitary fibrous tumors of the pleura mostly arise from the visceral pleura; they typically occur in the sixth and the seventh decades of life and have no gender predilection [2]. Unlike mesothelioma, SFTP have no association with exposure to asbestos. 

Solitary fibrous tumors of the pleura usually presents as an asymptomatic mass, as was the case with our patient, who had an incidental finding after the motor vehicle accident. However, SFTP may present with nonspecific symptoms, such as chest pain, cough, and dyspnea [2-3]. Most cases are benign but 20% of the time the tumor may be malignant [3]. Paraneoplastic syndromes associated with this tumor are hypertrophic osteoarthropathy and hypoglycemia [4]. 

On imaging, SFTP is seen as a sharply circumscribed pleural mass, as was seen with the patient discussed above. Occasionally, however, it may present with calcification, hemorrhage, and central necrosis [3]. 

On histological examination, patients with SFTP have cells that are elongated and spindle shaped. These cells are haphazardly arranged and separated by bands of collagen, forming a pattern often known as the “patternless pattern” [5]. An immunohistochemical analysis of the biopsy sample is used to differentiate SFTP from other pleural tumors. SFTP, in specific, tests positive for vimentin, CD 34 and/or BCL-2 and is nonreactive to desmin and S-100 [5]. Our patient’s biopsy sample had spindle-shaped cells, which stained positive for vimentin and CD-34 -- this was enough to confirm that she had SFTP. 

Consistent with what we observed in our patient, most SFTP are benign. Some, however, may be malignant; histologic features associated with malignancy are high mitotic activity, increased pleomorphism, and necrosis [6]. The most common treatment modality for both benign and malignant SFTP is surgical resection, which was also performed on our patient. In general, though, malignant tumors have a higher recurrence rate even after complete resection [7]. Due to the risk of recurrence of 10%-30%, annual chest radiographs are recommended [8]. 

While surgical resection continues to be the mainstay of treatment for SFTP [8], research is currently underway to understand the use of antiangiogenic agents for treatment purposes [9].

## Conclusions

Solitary fibrous tumors of the pleura are rare pleural tumors that clinicians should be aware of. Most patients may be asymptomatic and so the tumors might be found incidentally, hence it is important to differentiate any pleural tumors picked up on chest imaging from benign entities. Further research is awaited in terms of alternative treatments to surgical resection; such as antiangiogenic agents. Clinicians should also keep in mind the recurrent nature of the tumor and thus follow-up regularly.
